# Complete Genome Sequence of *Bacillus methylotrophicus* Strain B25, a Potential Plant Growth-Promoting Rhizobacterium

**DOI:** 10.1128/genomeA.00058-16

**Published:** 2016-03-10

**Authors:** Jonathan Gerbore, Aline Brutel, Arnaud Lemainque, Barbara Mairey, Claudine Médigue, David Vallenet, Francois Lefort, Damien Grizard

**Affiliations:** a BIOVITIS, Saint-Etienne-de-Chomeil, France; b Commissariat à l’Energie Atomique et aux Energies Alternatives (CEA), Institut de Génomique, Genoscope, Évry, France; c CNRS-UMR8030, Évry, France; d Université d’Evry Val d’Essonne, Évry, France; e Plants and Pathogens Group, Research Institute Land Nature and Environment, hepia, HES-SO University of Applied Sciences and Arts Western Switzerland, Geneva, Switzerland

## Abstract

The complete genome of *Bacillus methylotrophicus* strain B25, isolated in Switzerland, was sequenced. Its size is 3.85 Mb, and several genes that may contribute to plant growth-promoting activities were identified *in silico*.

## GENOME ANNOUNCEMENT

*Bacillus methylotrophicus* strain B25 (formerly *Bacillus amyloliquefaciens* subsp. *plantarum*) (1) is a Gram-positive bacterium isolated in Switzerland from the inner wood tissues of a decaying *Platanus* x *acerifolia* tree. Bacterial strains belonging to this clade are plant-associated microorganisms known to have beneficial effects on their hosts, such as growth promotion and health enhancement (2–4). The complete genome sequence of *B. methylotrophicus* strain B25 is reported here and will provide genomic information on the specific properties of this strain.

DNA was extracted with the GenElute bacterial genomic DNA kit (Sigma-Aldrich), according to the manufacturer’s instructions. Two libraries were constructed for sequencing at Genoscope. First, genomic DNA was fragmented, and inserts between 300 and 600 nucleotides (nt) were selected to construct a paired-end indexed library. Second, genomic DNA was fragmented, and inserts of around 8 kb were selected to construct a mate-paired indexed library. These libraries were loaded on an Illumina MiSeq sequencing device flowcell and sequenced on paired-ends, at 300 nt in length.

The reads from the paired-end library were merged with a homemade program and were assembled with mate-paired data using Newbler (overlap layout consensus [OLC]) (Roche). To reduce the number of undetermined bases, GapCloser was used (http://soap.genomics.org.cn/soapdenovo.html).

The remaining gaps were then covered using PCRs and specific primers for gap edges. The PCR fragments were sequenced by primer walking. The whole-assembly data were integrated into the MicroScope platform (http://www.genoscope.cns.fr/agc/microscope) for automatic annotation (5).

The complete genome of *B. methylotrophicus* strain B25 contains a 3,854,619-bp circular chromosome and an 8,138-bp plasmid. The genome contains 3,679 coding sequences (CDSs), with an average length of 934 bp, 21 rRNAs, and 68 tRNAs, and has a G+C content of 46.7%. The annotation predicted gene clusters coding for nonribosomal peptide synthetases (NRPS) and polyketide synthases (PKS). Among all predicted protein-coding genes, the strain B25 genome includes interesting genes involved in bacterium-plant interactions and plant growth promotion activities, i.e., the bacillibactin siderophore, an operon required for biofilm formation (*epsA-0*), and a phytate-degrading enzyme (phytase) (6). Gene resistance analysis was conducted with the CARD system (http://arpcard.mcmaster.ca/) (7). The B25 genome contains only natural resistance genes conserved at the species level. Gene content comparisons and synteny conservations indicate that the closest neighbors of B25 are *B. methylotrophicus* strains FZB42 (6), IT-45 (6), LFB112 (8), and Y2 (9). Further studies and genome comparisons of *B. methylotrophicus* strain B25 will enable the elucidation of the mechanisms supporting its plant-benefiting properties.

### Nucleotide sequence accession numbers.

This whole-genome shotgun project has been deposited at DDBJ/EMBL/GenBank under the accession numbers LN999829 and LN999830.
